# Incidence and factors associated with mortality in 2,476 patients with variant angina in Korea

**DOI:** 10.1038/srep46031

**Published:** 2017-04-06

**Authors:** Hack-Lyoung Kim, Jayeun Kim, Hyun Joo Kim, Woo-Hyun Lim, Jin Yong Lee

**Affiliations:** 1 Division of Cardiology, Department of Internal Medicine, Boramae Medical Center, Seoul, Korea; 2 Seoul National University College of Medicine, Seoul, Korea; 3 Institute of Health and Environment, Seoul National University, Seoul, Korea; 4 Department of Nursing Science, Shinsung University, Dangjin, Korea; 5 Public Health Medical Service, Boramae Medical Center, Seoul, Korea; 6 Institute of Health Policy and Management, Medical Research Center, Seoul National University, Seoul, Korea

## Abstract

This study investigated the incidence and risk factors of mortality in 2,476 patients with variant angina (VA) using the National Health Insurance Service–National Sample Cohort between 2004 and 2011. The risk factors of all-cause and cardiac mortality were investigated using Cox proportional hazards model. Most patients (69.5%) were less than 65 years and 42.9% were women. During the median follow-up duration of 4.9 years, there were 178 (7.2%) and 95 (3.8%) cases of all-cause and cardiac mortality, respectively. Older age, hypertension, diabetes mellitus, poor medication adherence, low household income and tertiary teaching hospitals were independent predictors for all-cause mortality, while older age, hypertension, low household income and tertiary teaching hospitals were independent predictors for cardiac mortality. In conclusion, our findings suggest that traditional risk factor control and continued medication are important to improve VA outcomes, and that household income-level factors should be considered in the assessment of risk of VA patients.

Variant angina (VA) is characterized by chest pain that is not associated with exertion, and is frequently accompanied by transient ST segment elevation on electrocardiogram^1^,^2^,^3^. Reversible coronary artery spasm has been established as the cause of VA^4^,^5^. Vasodilators are effective in relieving coronary artery spasm^6^,^7^,^8^,^9^,^10^; thus, as long as VA patients are taking vasodilators, their prognoses are better than those with atherosclerotic coronary stenosis^11^,^12^,^13^. However, sometimes, severe coronary spasm causes acute myocardial infarction (AMI) and fatal arrhythmia such as ventricular tachycardia, ventricular fibrillation and complete atrioventricular block, and subsequent cardiac arrest and death^11^,^14^,^15^. Even though some studies have investigated the outcomes and prognostic factors of patients with VA^6^,^7^,^11^,^12^,^13^,^14^,^15^,^16^,^17^,^18^,^19^,^20^,^21^,^22^, they had several weak points; first, sample sizes were relatively small and/or follow-up durations were short in most of those studies, therefore they would potentially have generalization problem; second. some of studies were performed several decades prior^6^,^7^,^13^,^14^,^15^, and the current VA treatment strategy using newer VA treatments such as K_ATP_ channel agonist (nicorandil)^23^,^24^, was not available for use in those studies. Therefore, investigations on VA with large subject numbers and long-term clinical follow-up duration are needed to better understand and manage this disease. Moreover, it would be helpful for such investigations to include more recent clinical practices for VA.

The purpose of this study was to determine the long-term mortality in 2,476 patients who had VA as a primary diagnosis between 2004 and 2011, and to identify the prognostic factors.

## Results

### Baseline characteristics of study subjects

The baseline characteristics of study subjects are shown in Table 1. In all subjects (n = 2,476), most patients (69.5%) were young and middle aged (<65 years), and 42.9% were female. About two thirds of subjects had hypertension (65.2%), 21.5% diabetes mellitus, and 37.8% dyslipidemia. Approximately half (53.5%) of the subjects showed good adherence to VA medications. Most (82.6%) of the subjects were treated in tertiary teaching hospitals, and 96.8% of the subjects had health insurance. About half (45.6%) of the subjects lived in metropolitan areas. Although there were some variations in the incidence of clinical variables as well as the number of VA patients each year, no specific trends were observed from 2004 to 2011.

### Mortality rates

**Figure 1:**
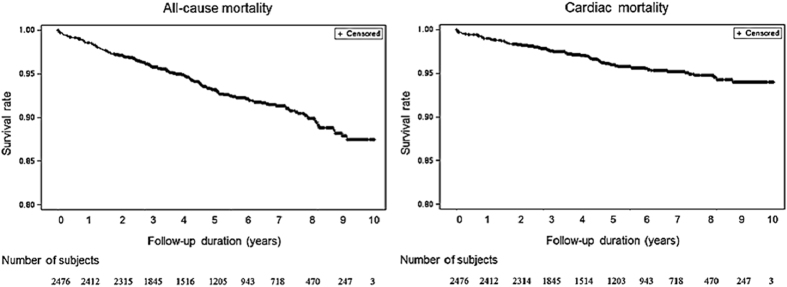
Survival curves in patients with variant angina.

All-cause and cardiac mortality rates are shown in Kaplan-Meyer curves (Fig. 1). During the median follow-up duration of 1,791 days (interquartile range, 1,089–2,673 days), there were 178 (7.2%) and 95 (3.8%) cases of all-cause mortality and cardiovascular mortality, respectively (Table 2). The annualized all-cause mortality and cardiovascular mortality rates of VA patients were 1.38% and 0.73%, respectively. The all-cause mortality rates were 11.2% in 2004, and 3.5% in 2011, and cardiovascular mortality rates were 4.7% in 2004 and 1.4% in 2011 with median follow-up duration of 3,431 days in 2004 and 864 days in 2011.

### Mortality predictors

The independent predictors of all-cause and cardiovascular mortality are shown in Table 3. Older age (15–49 versus ≥65 years: hazard ratio [HR], 10.83; 95% confidence interval [CI], 5.85–20.02), hypertension (HR, 1.67; 95% CI, 1.11–2.52), diabetes mellitus (HR, 1.39; 95% CI, 1.02–1.91), poor medication adherence (HR, 1.90; 95% CI, 1.40–2.57), tertiary teaching hospitals (HR, 2.00; 95% CI, 1.25–3.22), and low household income (highest versus lowest: HR, 1.52; 95% CI, 1.00–2.30) and were independent predictors of all-cause mortality. Older age (15–49 years versus ≥65 years: HR, 6.64; 95% CI, 3.20–13.77), hypertension (HR, 1.92; 95% CI, 1.07–3.46), tertiary teaching hospitals (HR, 2.72; 95% CI, 1.30–5.66), and low household income (highest versus lowest: HR, 1.80; 95% CI, 1.04–3.11) were independent predictors of cardiovascular mortality. Poor medication adherence showed marginal statistical significance as a risk factor of cardiovascular mortality (HR, 1.50; 95% CI, 0.99–2.28).

### Interactions between medication adherence and clinical variables

Interactions between poor medication adherence and mortality by age, sex, comorbidities, type of medical center, type of health security, household income, and region are shown in Table 4. There were no interaction in associations between poor medication adherences and mortality by sex, comorbidities, type of medical center, type of health security, household income, and region (*P* < 0.1 for each). Old age (aged ≥65 years) was the only factor having interaction with poor medication adherence in the prediction of total and cardiac mortality (*P* = 0.005 for each).

### Factors associated with good medication adherence

Independent factors associated with good medication adherence are shown in Table 5. Older age (15–49 versus ≥65 years: OR, 1.32; 95% CI, 1.16–1.50), male sex (male versus female, OR, 0.89; 95% CI, 0.82–0.97), hypertension (OR, 1.34; 95% CI, 1.23–1.47), diabetes mellitus (OR, 1.14; 95% CI, 1.04–1.24), non-tertiary teaching hospitals (non-tertiary teaching hospital versus tertiary teaching hospital, OR, 0.72; 95% CI, 0.64–0.80), and non-metropolitan region (OR, 1.12; 95% CI, 1.03–1.21) were independent factors associated with good medication adherence.

## Discussion

The results of our study of a large population of VA patients with a median follow-up of 1,791 days (=4.9 years) showed that the all-cause mortality of VA patients was 7.2% (annualized all-cause mortality rate = 1.38%) and cardiovascular mortality was 3.8% (annualized cardiovascular mortality rate = 0.73%). In VA patients, older age, hypertension, diabetes mellitus, poor medication adherence and low household income were independent risk factors of all-cause mortality, while older age, hypertension and low household income were independent risk factors of cardiovascular mortality. To the best of our knowledge, this is the largest study in the literature with a long follow-up period that shows the mortality rates and predictors in VA patients. Of additional interest, the independent association between low household income and poor VA outcomes was a meaningful and novel finding that has not been addressed in the literature to date.

The incidence of VA mortality has been reported by several studies^6^,^7^,^11^,^12^,^13^,^14^,^15^,^16^,^17^,^18^,^19^,^20^,^21^,^22^. However, those reports were limited in size and/or duration of follow-up. Bory *et al*.^6^ investigated 277 VA patients in France with a median follow-up of 7.4 years, and found all-cause and cardiac mortality rates of 7.2% and 3.6%, respectively, similar to our findings. A more recent study performed in Europe with 270 VA patients showed that 11.1% of patients died of cardiac causes after 3 years of follow-up^12^. The relatively high incidence of cardiac death in those patients was likely because of all the study patients had combined organic coronary stenosis. Indeed, none of the 76 VA patients without culprit coronary lesions had cardiac death in the same study^12^. Similarly, Figueras *et al*.^18^ reported a very low rate (7.0%) of VA-associated cardiac mortality in 273 VA patients without significant organic coronary stenosis during 12 years of follow-up. A study of Japanese nationwide registry data of 1,244 VA patients showed that the incidence of all-cause mortality and cardiac mortality during 2.7 years of follow-up was 1.3% and 0.3%, respectively^25^. Korean registry data including 2,129 patients with vasospasm revealed that the 2-year incidence of cardiac death was 0.9%^19^. Considering our study’s considerably longer follow-up duration (median 4.9 years), the rates of cardiac mortality of the previous Korean study could be expected to be similar to ours when the follow-up duration was increased about five fold. Our present study is the largest (n = 2,476) thus far with a long follow-up period (median, 4.9 years). Discrepancies among studies are mainly attributed to differences in study populations, diagnostic and management methods and length of follow-up. In general, VA prognosis has been reported to be worse in Western countries than in Asian countries^24^. Some previous studies that were performed several decades ago, demonstrated that annualized mortality rates of VA patients were >5%^7^,^22^, much higher than recent studies including ours.

Our results showed that VA mortality rates continuously decline from 2004 to 2011. However, this result should be interpreted cautiously because of lead-time bias. Lower mortality rates might be associated with shorter follow-up duration in patients more recently diagnosed with VA.

Several clinical parameters have been known to be risk factors of poor VA outcome. These include cigarette smoking, combined organic coronary stenosis and multi-vessel spasm^20^. Common cardiovascular risk factors including advanced age, hypertension and diabetes mellitus were independently associated with mortality in our study. Consistent with this finding, a previous study showed that systemic hypertension was a predictor of major coronary evens in VA patients in a multivariable analysis^6^. Another study also showed that more advanced age, hypertension and dyslipidemia were independent predictors of sudden cardiac death in VA patients^16^. However, a contradicting result indicated that traditional risk factors such as hypertension, diabetes mellitus and hypertension did not affect VA prognosis^26^. Further studies focused on this issue should clarify the association between classical cardiovascular risk factors and VA outcomes.

Patients’ adherence to medications is very important for the reduction of the frequency and severity of VA attacks. Abrupt cessation of medications is frequently associated with a more severe form of VA attack, as a rebound phenomenon^27^,^28^. In agreement with this finding, our study also showed that poor adherence to vasodilator therapy was independently associated with all-cause mortality in VA patients. We also showed that most of clinical variables did not have interactions with poor medication adherence in the prediction of mortality. Considering that half of patients had poor medication adherence in our study, identification of an effective medical strategy for improving medication adherence may significantly improve VA outcomes. Younger age, female sex and absence of comorbidities including hypertension and diabetes mellitus were independently associated with poor medication adherence in our study. Physicians should recognize the potential hazards of vasodilator withdrawal, and emphasize the importance of regular medications especially in young female VA patients without hypertension and diabetes mellitus to improve their outcomes as well as reduce VA attacks.

The influence of socioeconomic status on VA prognosis has not been reported. We found that lower household income was associated with an increased risk of VA mortality. The inverse relationship between socioeconomic status and other ischemic heart disease has been well described^29^,^30^. As a consistent finding, our study also showed that low household income was independently associated with increased all-cause and cardiac mortality in VA patients. Patients with lower household income may not receive appropriate prevention and management of cardiovascular disease. Risk factors are more likely to be poorly controlled, and seeking of care is delayed. Indeed, it has been shown that people with lower socioeconomic status have more unfavorable risk factors such as smoking, high blood pressure and elevated cholesterol. They also undergo fewer cardiac procedures, take fewer medications and utilize less post hospital discharge care and follow-up^31^. Socioeconomic status should be considered as an important factor in the assessment of risk in patients with VA, and more research is needed to further characterize this issue.

Our results showed that VA patients managed in tertiary teaching hospitals had worse prognosis than those in non-tertiary teaching hospitals. This may be due to differences in case mix, with more severe and complicated cases were transferred and managed in these hospitals.

Aside from the retrospective nature of this study, several limitations should be considered. First, errors from possible coding inaccuracy may be the main limitation of such health insurance-based database^32^. Serious VA-associated complications such as cardiac arrest, AMI or ventricular arrhythmia could be a primary diagnosis, and the VA diagnosis could be masked or missed. Second, methods used for VA diagnosis were not available. Third, information on other risk factors for VA outcomes such as cigarette smoking, combined atherosclerotic coronary stenosis, and multi-vessel spasm was lacking^20^. Additionally, information on medications other than vasodilators was lacking in our study. In particular, antiplatelets, statin and renin-angiotensin system blockers might also have an impact on patients’ survival. Fourth, the follow-up periods varied in individual patients depending on the date that VA was diagnosed, and some morality cases may not have been recorded when the patients were not being monitored. Despite these potential limitations, the present study has important clinical implications because a real-world large sample sized data from a national database was used, and without selection bias.

In conclusion, the present nationwide study showed that the annualized all-cause mortality and cardiac mortality rates of Korean VA patients were 1.38% and 0.73%, respectively. Older age, hypertension, diabetes mellitus, poor medical adherence and low socioeconomic status were associated with VA mortality. These findings provide additional evidence that traditional risk factor control and continued medication are important to improve VA outcomes. In addition, our study suggests that household income-level factors should be considered in the assessment of risk of VA patients.

## Methods

### Data sources

We used 2002–2013 data from the National Health Insurance Service–National Sample Cohort (NHIS-NSC), a population-based cohort established by the National Health Insurance Service (NHIS) in South Korea^33^. Korea has been operating the mandatory universal healthcare coverage system through the NHIS^33^. Every Korean citizen must be covered by the mandated universal healthcare system from birth to death, either National Health Insurance (NHI) for general populations or Medical Aid (MA) for the vulnerable, which is being managed by NHIS^34^. Financially, NHI is mainly funded by NHI premiums gathered from beneficiaries and receiving some government subsides, while MA is funded by taxes. MA is the only option available for the Korean populations who are unable to pay the NHI premium (e.g., low-income families), similar to Medicaid in the United States^34^. As of 2011, there were 50,908,646 citizens of Korea, consisting of 49,299,165 (96.8%) NHI beneficiaries and 1,609,481 (3.2%) MA recipients^35^. NHIS provides benefits for prevention, diagnosis, and disease and injury treatment, as well as rehabilitation, births, deaths, and health promotion. NHIS gathers, maintains, and stores all medical claims in a database consisting of diagnoses, treatments, healthcare utilization, and prescriptions^33^. For example, NHIS had 1.3 billion medical claims from Korean citizens in 2011^34^. NHIS established the National Health Insurance Service–National Sample Cohort (NHIS-NSC), a population-based cohort database, whose sole purpose is to provide public health researchers and policy makers with representative and useful information regarding citizens’ healthcare utilizations and health statu^33^.

**Figure 2:**
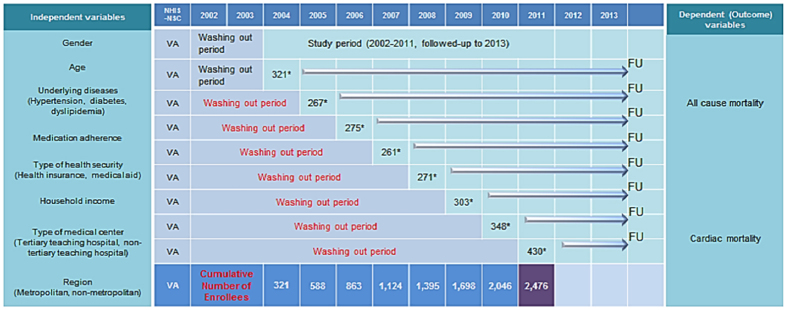
Schematic representation of enrolment and follow-up of study subjects. *Number of new enrollees in each consecutive year from 2004 to 2011 applying at least 2 year to maximum 9 year washing out period. VA, variant angina; FU, follow-up.

We used 2002–2013 data from NHIS-NSC, released by NHIS in 2014. This cohort data contains 1,025,340 people (as of 2002, approximately 2.2% of the entire Korean population) who were randomly selected for representing the entire Korean population^33^. In order to make randomized cohort samples, NHIS used probabilistic sampling to represent an individual’s total annual medical expenses within each of 1476 strata defined by age, sex, eligibility status (employed or self-employed), and income level (20 quartiles for each eligibility status and MA recipients) combinations via proportional allocation from the 46,605,433 Korean residents in 2002^33^,^36^,^37^. In particular, NHIS-NSC was designed to be a semi-dynamically constructed cohort database; the cohort has been followed up to either the time of the participant’s disqualification from receiving health services due to death or emigration or until the end of the study period, whereas samples of newborn infants are included annually^33^,^36^,^37^. The database contains eligibility and demographic information regarding NHI beneficiaries as well as data on MA recipients, medical bill details, medical treatment, disease histories, and prescriptions^33^,^36^,^37^. NHIS-NSC data accessed in the current study included demographic information, including sex, age, residence regions, household income, and type of health security. In addition, inpatient and outpatient medical care utilization information for primary and subsidiary causes, including date of service, and drugs prescribed were included. The causes of medical care utilization or death were recorded under the International Classification of Diseases, 10^th^ reversion (ICD-10). We included study subjects whose (1) primary cause of medical service usage was ‘I201 ’ or (2) subsidiary cause of ‘I201’ and cause of death of ‘I’, with no prior medical service usage under ‘I201’, either primary or subsidiary, before the time of inclusion in the study. For the onset of VA, we established 2002 to 2003 as the washing-out period, and excluded patients with any treatment or diagnosis history of VA during this period. To specifically detect new VA patients, we established a washing-out period from at least two years to nine years. Figure 2 shows the detailed information about how new enrollees were selected in each consecutive year from 2004 to 2011 after applying at least 2 year to maximum to 9 year washing out period. Using this model, enrollees in 2004 were the new onset VA patients with no prior diagnosis of VA during 2002–2003. (specifically, 267 enrollees in 2005 were new patients who were never diagnosed or treated for VA during 2002–2004 and 430 enrollees in 2011 were new patients with no record of VA during 2002–2010). Accordingly, patients with VA onset in 2004 would have a maximum follow up duration of nearly 10 years, and patients with VA onset in 2011 would have less follow up duration, but the duration would be >2 years. Follow-up for all VA patients was completed at the end of 2013 (Fig. 2).

### Baseline clinical data

The subjects’ ages were grouped into 15–49, 50–64, and ≥65 years. Information on cardiovascular risk factors including hypertension (ICD-10 I100, I101 and I109), diabetes mellitus (ICD-10 E10–E14), and dyslipidemia (ICD-10 E78) was obtained. To assess medication adherence, all drugs administered to patients for a year after onset of VA were reviewed. In order to evaluate medication adherence, we used medication possession ratio (MPR), a ratio of total days’ supply to numbers of days of study participation per participant^38^,^39^. Good medication adherence was defined as a cumulative medication adherence of more than 80% (approximately annually 300 days) per study period among patients with VA prescribed in a given period and included data only on VA medication prescription dispensed from pharmacies^40^,^41^. In this study, VA medication refers vasodilators including diltiazem, verapamil, nitrate, nicorandil, and molsidomin. Household income was stratified into three groups according to income quintile: quintile 1–3, quintile 4–8, and quintile 9–10 groups. Metropolitan areas referred to seven major cities (Seoul, Busan, Daegu, Incheon, Gwangju, Daejeon, and Ulsan) in South Korea.

### Mortality data

NHIS-NSC records were merged with certified date of death from the Statistics Korea, and the cause of death and death date were recorded. Cardiovascular death was defined as a case with circulatory cause of death (ICD-10 I00-I99). For the death date, information on only the year and month of death was obtained, and the following methods were used to calculate the follow-up duration until death: if the date of final medical care utilization was in the year and month as that of death, we set the death date by adding a nursing period (days) to the start of the date of care. In case the date of final medical care utilization was not in the same year or month as that of death, we set the death date on the middle of the year and month.

### Statistical analysis

Descriptive statistics of the study population are presented as n (%) for dichotomous variables and mean ± standard deviation for continuous variables. Cox proportional hazards model was used to investigate the association of candidate risk factors with mortality. For censored cases, we set the end of the follow-up point as the last date of medical care utilization, since it was certain that these patients were alive when they utilized the medical care services. Age, sex, cardiovascular risk factors, medication adherence, type of medical center, type of health security, household income, and residence region were considered as potential confounders and were adjusted during multivariable Cox regression analysis. For the interaction analyses, we fitted cross-product terms between poor medication adherence and clinical variables in order to investigate whether the effect of poor medication adherence on mortality was changed by other clinical variables. Multiple binary logistic regression analysis was performed to investigate independent factors for good medication adherence. Age, sex, cardiovascular risk factors, type of medical center, type of health security, household income, and residence region were considered as potential confounders and were adjusted during Multiple binary logistic regression analysis. All analyses were conducted using SAS version 9.4 (SAS Institute Inc., Cary, NC, USA) and *P* < 0.05 was regarded as statistically significant.

### Ethical Statement

Ethical approval was waived in this study by the Institutional Review Board (IRB) of Boramae Medical Center (Seoul, Korea) (#IRB No. 07-2016–13). Informed consent was not obtained because patient records and information were anonymized and de-identified prior to analysis.

## Additional Information

**How to cite this article:** Kim, H.-L. *et al*. Incidence and factors associated with mortality in 2,476 patients with variant angina in Korea. *Sci. Rep.* **7**, 46031; doi: 10.1038/srep46031 (2017).

**Publisher's note:** Springer Nature remains neutral with regard to jurisdictional claims in published maps and institutional affiliations.

## Figures and Tables

**Table 1:** Baseline characteristics of study subjects.

Variable Year	Total (n = 2,476)	2004 (n = 321)	2005 (n = 267)	2006 (n = 275)	2007 (n = 261)	2008 (n = 271)	2009 (n = 303)	2010 (n = 348)	2011 (n = 430)
Age group									
15–49 years	644 (26.0)	96 (29.9)	79 (29.6)	73 (26.5)	81 (31.1)	65 (24.0)	79 (26.1)	76 (21.8)	95 (22.1)
50–64 years	1,076 (43.5)	133 (41.4)	111 (41.6)	124 (45.1)	111 (42.5)	116 (42.8)	138 (45.5)	167 (48.0)	176 (40.9)
≥65 years	756 (30.5)	92 (28.7)	77 (28.8)	78 (28.4)	69 (26.4)	90 (33.2)	86 (28.4)	105 (30.2)	159 (37.0)
Female sex	1,062 (42.9)	121 (37.7)	126 (47.2)	114 (41.5)	105 (40.2)	132 (48.7)	128 (42.2)	146 (42.0)	190 (44.2)
Cardiovascular risk factors									
Hypertension	1,615 (65.2)	229 (71.3)	170 (63.7)	198 (72.0)	163 (62.5)	178 (65.7)	189 (62.4)	231 (66.4)	257 (59.8)
Diabetes mellitus	533 (21.5)	53 (16.5)	52 (19.5)	62 (22.5)	66 (25.3)	51 (18.8)	57 (18.8)	96 (27.6)	96 (22.3)
Dyslipidemia	937 (37.8)	102 (31.8)	92 (34.5)	104 (37.8)	80 (30.7)	93 (34.3)	142 (46.9)	138 (39.7)	186 (43.3)
Good medication adherence	1,325 (53.5)	208 (64.8)	151 (56.6)	149 (54.2)	113 (43.3)	135 (49.8)	171 (56.4)	188 (54.0)	210 (48.8)
Tertiary teaching hospital	2,044 (82.6)	254 (79.1)	221 (82.8)	231 (84.0)	199 (76.2)	228 (84.1)	244 (80.5)	306 (87.9)	361 (84.0)
Type of medical security									
Health insurance	2,398 (96.8)	320 (99.7)	264 (89.9)	274 (99.6)	258 (98.9)	258 (95.2)	290 (95.7)	347 (99.7)	387 (90.0)
Medical aid	79 (3.2)	1 (0.3)	3 (1.1)	1 (0.4)	3 (1.1)	13 (14.8)	13 (4.3)	1 (0.3)	43 (10.0)
Household income									
0–3 quintile (lowest)	519 (21.0)	50 (45.6)	49 (18.4)	56 (20.4)	47 (18.0)	70 (25.8)	61 (20.1)	61 (17.5)	125 (29.1)
4–8 quintile (middle)	1,135 (45.8)	147 (45.8)	125 (46.8)	131 (47.63)	121 (46.4)	123 (45.4)	142 (46.9)	168 (48.3)	178 (41.4)
9–10 quintile (highest)	822 (33.2)	124 (38.6)	93 (34.8)	88 (32.0)	93 (35.6)	78 (28.8)	100 (33.0)	119 (34.2)	127 (29.5)
Living in metropolitan	1,128 (45.6)	138 (43.0)	120 (44.9)	108 (39.3)	121 (46.4)	128 (47.2)	151 (49.8)	160 (46.0)	202 (47.0)

Values are represented as n (%).

**Table 2:** All-cause and cardiovascular mortality.

Variable Year	Total	2004	2005	2006	2007	2008	2009	2010	2011
Subject number	2476	321	267	275	261	271	303	348	430
Follow-up duration, median (interquartile range), days	1,791 (1,089–2,673)	3,431 (3,295–3,560)	2,982 (2,913–3,137)	2,650 (2,565–2,764)	2,285 (2,188–2,412)	1,934 (1,830–2,049)	1,539 (1,466–1,680)	1,169 (1,096–1,297)	864 (747–982)
All-cause mortality, n (%)	178 (7.2)	36 (11.2)	31 (11.6)	27 (9.8)	12 (4.6)	18 (6.6)	16 (5.3)	23 (6.6)	15 (3.5)
CV mortality, n (%)	95 (3.8)	15 (4.7)	17 (6.4)	19 (6.9)	8 (3.1)	8 (3.0)	12 (4.0)	10 (2.9)	6 (1.4)

CV, cardiovascular.

**Table 3:** Independent predictors for all-cause and cardiovascular mortality.

Variable	Hazard ratio (95% confidence interval)	
	All-cause mortality	Cardiovascular mortality
Age		
15–49 years	1.00	1.00
50–64 years	1.89 (0.97–3.65)	1.14 (0.51–2.57)
≥65 years	10.83 (5.85–20.02)	6.64 (3.20–13.77)
Sex		
Male	1.00	1.00
Female	0.78 (0.58–1.06)	1.08 (0.72–1.63)
Underlying diseases		
Hypertension, yes	1.67 (1.11–2.52)	1.92 (1.07–3.46)
Diabetes mellitus, yes	1.39 (1.02–1.91)	1.20 (0.77–1.88)
Dyslipidemia, yes	0.92 (0.67–1.25)	0.74 (0.48–1.14)
Medication adherence		
Good	1.00	1.00
Not good	1.90 (1.40–2.57)	1.50 (0.99–2.28)
Type of medical center		
Non-tertiary hospitals	1.00	1.00
Tertiary teaching hospitals	2.00 (1.25–3.22)	2.72 (1.30–5.66)
Type of health security		
Health insurance	1.00	1.00
Medical aid	1.18 (0.50–2.83)	0.55 (0.13–2.37)
Household income		
9–10 quintile (highest)	1.00	1.00
4–8 quintile (middle)	1.34 (0.94–1.90)	1.32 (0.81–2.14)
0–3 quintile (lowest)	1.52 (1.00–2.30)	1.80 (1.04–3.11)
Region		
Metropolitan	1.00	1.00
Non-metropolitan	0.96 (0.71–1.29)	1.03 (0.68–1.56)

**Table 4:** Interactions between non-medical adherence and clinical variables.

Variable	Total mortality		Cardiac mortality	
	HR (95% CI)	Interaction *P*	HR (95% CI)	Interaction *P*
Age				
15–49 years	0.41 (0.12–1.37)		0.24 (0.05–1.15)	
50–64 years	1.21 (0.63–2.35)	0.122	0.49 (0.16-1.49)	0.462
≥65 years	2.47 (1.74–3.52)	0.005	2.44 (1.50–3.97)	0.005
Sex				
Male	2.06 (1.37–3.08)		1.34 (0.74–2.45)	
Female	1.72 (1.10–2.69)	0.560	1.66 (0.94–2.95)	0.610
Hypertension				
Hypertension, no	2.03 (0.93–4.41)		1.31 (0.45–3.84)	
Hypertension, yes	1.88 (1.35–2.60)	0.855	1.53 (0.98–2.40)	0.791
Diabetes				
Diabetes mellitus, no	1.83 (1.26–2.65)		1.24 (0.75–2.04)	
Diabetes mellitus, yes	2.04 (1.22–3.43)	0.732	2.35 (1.10–5.02)	0.161
Dyslipidemia				
Dyslipidemia, no	1.90 (1.30–2.78)		1.65 (1.00–2.73)	
Dyslipidemia, yes	1.89 (1.15–3.12)	0.989	1.22 (0.58–2.58)	0.508
Type of medical center				
Tertiary teaching hospitals	1.91 (1.39–2.64)		1.62 (1.05–2.50)	
Non-tertiary hospitals	1.79 (0.73–4.41)	0.891	0.67 (0.16–2.83)	0.250
Type of health security				
Health insurance	1.83 (1.34–2.49)		1.48 (0.97–2.26)	
Medical aid	5.24 (0.95–28.75)	0.234	2.81 (0.18–45.10)	0.655
Household income				
9–10 quintile (highest)	1.87 (1.10–3.18)		1.43 (0.64–3.20)	
4–8 quintile (middle)	1.81 (1.15-2.85)	0.927	1.54 (0.82-2.89)	0.961
0–3 quintile (lowest)	2.10 (1.15–3.83)	0.774	1.51 (0.72–3.17)	0.930
Region				
Metropolitan	2.03 (1.30–3.17)		1.64 (0.95-2.83)	
Non-metropolitan	1.80 (1.20–2.70)	0.697	1.34 (0.72–2.50)	0.630

HR; Hazard ratio, CI; Confidence interval.

**Table 5:** Independent factors associated with good medication adherence.

Variable	Odds ratio (95% confidence interval)
Age	
15–49 years	1.00
50–64 years	1.19 (1.07–1.33)
≥65 years	1.32 (1.16–1.50)
Sex	
Male	1.00
Female	0.89 (0.82–0.97)
Underlying diseases	
Hypertension, yes	1.34 (1.23–1.47)
Diabetes mellitus, yes	0.93 (0.84–1.03)
Dyslipidemia, yes	1.14 (1.04–1.24)
Type of medical center	
Non-tertiary hospitals	1.00
Tertiary teaching hospitals	0.72 (0.64–0.80)
Type of health security	
Health insurance	1.00
Medical aid	1.27 (0.97–1.64)
Household income	
0–3 quintile (lowest)	1.00
4–8 quintile (middle)	1.03 (0.92–1.16)
9–10 quintile (highest)	0.89 (0.79–1.01)
Region	
Metropolitan	1.00
Non-metropolitan	1.12 (1.03–1.21)
